# Wavelength-Specific Behavior of the Western Flower Thrips (*Frankliniella occidentalis*): Evidence for a Blue-Green Chromatic Mechanism

**DOI:** 10.3390/insects11070423

**Published:** 2020-07-09

**Authors:** Niklas Stukenberg, Markus Pietruska, Axel Waldherr, Rainer Meyhöfer

**Affiliations:** 1 Institute of Horticultural Production Systems, Section Phytomedicine—Applied Entomology, Leibniz Universität Hannover, 30419 Hannover, Germany; stukenberg@uni-bonn.de (N.S.); markus_pietruska@yahoo.de (M.P.); axel.waldherr@htp-tel.de (A.W.); 2 Institute of Crop Science and Resource Conservation, Agroecology and Organic Farming Group, University of Bonn, 53121 Bonn, Germany

**Keywords:** color preference, color vision, chromatic interaction, action spectra, light-emitting diode, LED, visual trap, monitoring

## Abstract

The western flower thrips (*Frankliniella occidentalis*) is a serious pest in horticulture, feeding on leaf tissue and floral resources. Blue and yellow sticky traps are commonly used for monitoring and control in greenhouses. The mechanisms underlying the color preferences are largely unknown. The use of light-emitting diodes (LEDs) is a promising approach to increase the attractiveness of visual traps and to study the color choice behavior in insects. The color preferences of *F. occidentalis* were systematically investigated in a series of choice experiments with several LEDs from the ultraviolet (UV) and visible spectral range. Blue LEDs were most attractive, followed by green, while only a moderate attractiveness of UV was observed. Blue and green were identified as two separate attractive ranges. When light from blue and green LEDs was mixed, the attractiveness decreased compared to its single components. In conclusion, *F. occidentalis* exhibits two different wavelength specific behaviors towards blue and green. Compelling indications are provided that these behaviors are controlled by two photoreceptors maximally sensitive in the blue and green range with an inhibitory chromatic interaction between both. Since the known UV sensitive photoreceptor could be confirmed, a trichromatic photoreceptor setup is suggested for *F. occidentalis*. For advanced plant protection strategies, the results offer several opportunities to optimize monitoring or even develop mass trapping devices.

## 1. Introduction

Many insect species are adapted to unique plant resources while others forage on multiple resources, i.e., leaves, flowers, and fruits. Well-known examples are thrips species, e.g., western flower thrips (*Frankliniella occidentalis*), feeding on leaf tissue as well as on floral resources like pollen and nectar. Although nutritional needs might change during the life cycle, color cues are, besides olfactory cues, important for detection of different plant characteristics. Visual-guided host plant detection is generally accepted as an indispensable binding link between potential olfactory orientation, which provides insufficient directional information, and selection upon host contact by herbivorous insects [1]. The importance of color cues for insect orientation has led to various applications in plant protection, since they allow the attraction without the involvement of any olfactory plant stimuli. For example, blue and yellow color traps are nowadays the most common method to detect a first infestation of thrips and whiteflies and to monitor the population development in vegetable and ornamental greenhouse crops [2,3,4]. The commercially available traps usually consist of a rectangular yellow or blue plate which is covered on both sides with a sticky glue so that the insects adhere to them [5,6]. The color traps are usually hung above the plant stand and the number of insects trapped is estimated weekly [7]. However, color traps have the disadvantage of reflecting a broad spectrum of light, which might not optimally fit to the photoreceptors and the visual processing system of the target insect. Furthermore, the intensity and thus attractiveness of the reflected radiation depends on the variable ambient light conditions, hence the sunlight intensity the trap is exposed to. The number of trapped insects is therefore quite variable and does not allow consistent conclusions about the actual population size in the crop stand [2,8]. As a rule, however, control measures should be implemented immediately if action thresholds are reached in order to prevent a high damage potential at an early stage. This requires a reliable catch quota, which is not always given by color as indicated above. Since the use of reflective color traps has the highlighted shortcomings, the use of light-emitting diodes (LEDs) alone or in addition to colored traps are a valid possibility to increase the attractiveness of visual traps [9,10]. LEDs emit narrow-bandwidth light independent of the ambient light, which can be individually controlled and combined. Color choice studies using stimuli with different broadband reflection patterns commonly do not clearly separate intensity and wavelength-dependent responses. Therefore, the use of adjustable narrow-bandwidth LEDs has also turned out to be a suitable tool to study the color choice behavior of insects [10,11,12,13]. With one exception [10], the analyses carried out on the color preference of *F. occidentalis* are based exclusively on the use of reflective color traps. The analyses of the several research groups shows also that in general blue, followed by yellow and white color traps, are preferred and can be used to trap *F. occidentalis*. Interestingly, the results differ with respect to the preferred colors, as sometimes yellow, sometimes blue, and sometimes white was documented as most attractive [2,7,14,15]. However, the different preferences can be partly attributed to the general use of color traps and their shortcomings as mentioned above.

The only available LED-based study on the color preference of *F. occidentalis* clearly shows blue LEDs being most attractive compared to green, confirming the known blue preference [10]. Based on this result, common blue sticky traps were equipped with single blue high-power LEDs with 445 nm peak wavelength, resulting in a significantly higher attractiveness.

The physiological basis for the visual perception of light are the photoreceptor cells in the insect compound eye containing the visual pigments. The only available electrophysiological study with *F. occidentalis* determined spectral efficiency peaks in the ultraviolet and the green range around 540 nm via electroretinogram recordings [16]. Results led to the conclusion that two different photoreceptor pigments are present in *F. occidentalis*. However, this finding contradicts the known blue preference because *F. occidentalis* responds strongly to a wavelength range, where only a low physiological sensitivity was observed. To our knowledge, no conclusive findings that could explain this discrepancy between behavioral and physiological data clarifying the underlying visual mechanisms have been provided so far.

Moreover, it must be noted that the blue preference in *F. occidentalis* is a quite uncommon phenomenon, as compared to the preference for yellow and green by other herbivorous insects [17,18]. The green-yellow preference is a familiar example for an innate response used for object detection, often referred to as a wavelength-specific behavior. An important mechanism for color perception is termed color opponency, where the outputs of several photoreceptors are compared by antagonistic neural interaction [19,20,21]. For aphids, whiteflies, and the pollen beetle, it was shown that the wavelength-specific settling behavior is controlled by such an inhibitory interaction of two overlapping photoreceptors, which are maximally sensitive for blue and green light, respectively. In this so called ‘blue-green opponency’ the signal from the blue receptor is inhibitory, while the signal from the green receptor is excitatory [13,22,23]. This mechanism facilitates the extraction of a chromatic signal that detects reflected long-wavelength light (green-yellow) associated with host plants and discriminates it from short- or broad-wavelength light. Due to the compelling evidence from several studies, blue-green opponency can be regarded as a universal and consistent mechanism in herbivorous insects. A choice study with the greenhouse whitefly using the mentioned advantages of LEDs contributed to the enlightenment of this mechanism, although physiological data was scarce. At equal intensities, a green LED proved to be most attractive, while this attraction was inhibited when combined with small amounts of blue LED light [13].

The sensitivity for ultraviolet radiation (UV) was clearly determined in a physiological study [17]. UV is known to be associated with skylight and affects the migratory behavior of *F. occidentalis* and other herbivorous insects. Studies with UV-absorbing plastic films showed that a UV-deficient environment was avoided in choice experiments and dispersal was reduced when thrips were released within such conditions [24]. UV reflection, which is a feature of flower petals, was also suggested as having an important positive effect on the attraction [2]. However, UV LEDs alone proved to be only moderately attractive in choice experiments under greenhouse conditions [10]. The knowledge on the combination of UV and colored LEDs regarding attractiveness and potential interactions are currently completely missing.

Therefore, the aim of the current study was to investigate the color preferences of *F. occidentalis* using LEDs in a choice arena under controlled conditions, following an already established approach [13]. The more specific objectives are to gain a better understanding of the visual perception regarding the behavioral action spectra, involved photoreceptors, and potential chromatic interactions. The obtained results will help to draw conclusions on the visual ecology and could help to develop enhanced biotechnological control strategies, which improve monitoring as well as mass trapping in greenhouse cropping.

## 2. Materials and Methods

### 2.1. Rearing of F. occidentalis

Thrips were permanently reared on bean plants (*Phaseolus vulgaris* L. var. Nanus) in cages (0.50 × 0.55 × 0.45 m) in a climate chamber (23 °C, 63% RH; Kälte Roter, Hannover, Germany). Thrips from that stock culture were transferred to glass jars (0.75 L) and bean pods and pollen were added for feeding, while a petri dish with quartz sand served as a pupation site. From these jars with unsynchronized thrips populations, bean pods with eggs only were transferred to new glass jars every 2–3 days for breeding of cohorts of synchronized populations. Bean pods were changed every 2–3 days, quartz sand was added after 7 days, and pollen was added after 14 days. All glass jars were kept in a climate cabinet (23 °C, 63% RH, 16:8 h L:D photoperiod; Rubarth Apparate GmbH, Hannover, Germany). It was already noted in an earlier study that adult thrips from the glass jar rearing barely responded in LED choice experiments [10]. For unknown reasons, the rearing of synchronized individuals from the jars for a single generation on bean plants turned out as a solution to generate flight active adults. Following the protocol of Berndt et al. (2004) [25] and Otieno et al. (2018), thrips on potted plants with microcosms were reared in the same climate chamber where the choice experiments took place (see experimental design section). Two weeks later, the plants were cut off from the pot and as a result of positive phototaxis, emerging adults from the pupation site in the substrate were trapped using photoeclectors (see Otieno et al., 2018 for further details). At least 120 individuals (sex ratio approx. 80% females: 20% males) were collected in Eppendorf tubes attached to the photoeclectors for each experimental trial.

### 2.2. Construction of Light Traps

**Figure 1:**
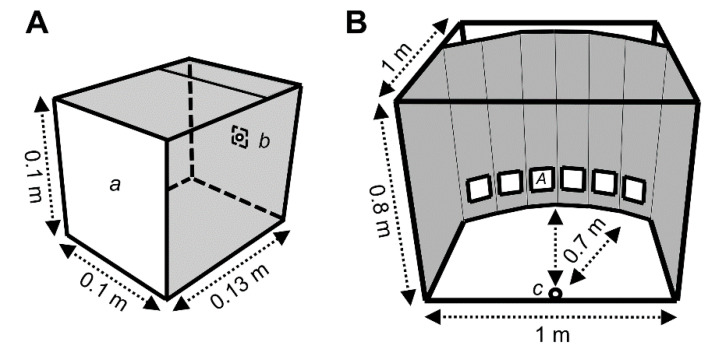
Schemes of LED trap and choice arena. (**A**) LED trap with acrylic glass screen front side (*a*) and LED panel backside (*b*). The inner side of the box was laminated with mirror film. (**B**) Choice arena with thrips release point (*c*) and position of LED traps (*A*). The background was black and the bottom was black-brown. In experiments with less than six traps, the not used openings on the background were covered with black foil.

The constructed light traps consist of housings (13 × 10 × 10 cm) made of polyvinylchloride plates (4 mm), as already used in previous studies [10,12,13]. Briefly, a colorless translucent acrylic glass plate with light scattering properties (Plexiglas^®^, LED 0M200 SC, Evonik Industries AG, Essen, Germany) is located at the front. At the back of the housing, aluminum panels (10 × 10 cm) hold the respective high-power (HP) LEDs (Figure 1A). In order to trap thrips, a standard wrapping film (polyethylene terephthalate) was stretched on the translucent screen and coated with insect glue (TEMMEN GmbH, Hattersheim, Germany).

### 2.3. Measurement and Adjustment of High-Power LEDs

**Figure 2:**
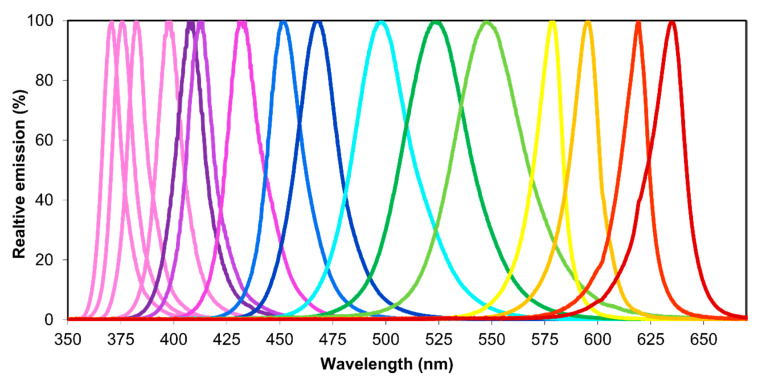
Spectra of high-power light-emitting diodes (LEDs) used. See Table 1 for specifications.

The specifications of all used LEDs are given in Table 1. Most LEDs were commercially available single-chip emitters but, as in a previous study, two specific multi-chip emitters were used which were equipped with additional cooling [13]. LEDs were operated as described in previous studies [10,12], allowing individual intensity adjustment. Using the same equipment and following the same protocols, all intensities were adjusted following Stukenberg and Poehing (2019). With one exception (see experimental details), LEDs and their combinations used in the choice tests were adjusted to equal intensities to obtain standardized, purely wavelength-dependent conditions. After adjusting intensities, the spectra of all LEDs were measured in complete darkness using the spectrometer AvaSpec-2048-2 (Avantes BV, Apeldoorn, The Netherlands) (Figure 2).

### 2.4. Experimental Design

All choice tests were done under controlled climatic conditions (23 °C; 65% relative humidity; 16:8-h L:D photoperiod) in a climate chamber (Johnson Controls, Essen, Germany). A flight cage with a base area of 1 × 1 m and a height of 0.8 m served as the arena (Figure 1B). While the side walls were covered with black plastic foil, the upper side of the cage consisted of a translucent foil. This ensured that the visible light (45 µmol m^−2^ s^−1^ on average) and UVA radiation (2 µmol m^−2^ s^−1^ on average) emitted by the fluorescent tubes could enter the cage. The LED traps were placed in up to six quadratic openings of a black cardboard box. Traps were positioned 7.5 cm above ground with 5 cm distance between each other. In experiments with less than six traps, the not used openings on the background were covered with black polyethylene foil. In multiple-choice experiments with five or four traps, the outer positions were not used. In dual-choice experiments, only the outer positions were used and the inner ones were covered. The order of LED traps was randomized for each experimental replicate on the six positions. LEDs were switched on prior to insect release for a minimum of 10 min to ensure a constant operating temperature. Thrips were always released at the same release point with 0.7 m distance to each of the traps from not more than three Eppendorf tubes, containing in total approx. 120 adult thrips. Experiments were terminated after 30 min and all thrips on the traps, inside the cage, or remaining in the Eppendorf caps were counted. All experiments were conducted between 9 am and 6 pm. Ten to 20 consecutive replicates were performed for each experiment.

### 2.5. Attractiveness of Different Wavelengths from the Ultraviolet to the Visible Spectral Range (307–635 nm)

Two experiments were conducted. While six color traps (UVA1, Blue2, Cyan, Green1, Yellow2, Red) were used in the first experiment, the blue LED (476 nm) was excluded in the second experiment to narrow down the attractiveness of the spectral range. All LEDs were adjusted to the intensity of the color trap Cyan (41.2 µmol m^−2^ s^−1^) in both sections. Twenty consecutive replicates were performed respectively.

### 2.6. Investigation of Thrips Visual Preference in the Green-Yellow (498–619 nm), Blue (413–498 nm), and Ultraviolet (371–413 nm) Spectral Ranges

Five experiments were conducted in total. In the first experiment, six LEDs (Cyan, Green1, Green2, Yellow1, Yellow2, and Amber) were used to examine the attractiveness of individual wavelengths in the green-yellow spectral range. All LEDs used were adjusted to the intensity of Yellow1 (27.6 µmol m^−2^ s^−1^). The two most attractive LEDs (Green2 and Yellow1) were then presented in a further dual-choice experiment. The following experiment focused on the examination of the blue spectral range. Compared to the first experiment, only five LEDs (Violet2, Violet3, Blue1, Blue2, and Cyan) were used. All LEDs were adjusted to the intensity of the Violet2 (51.4 µmol m^−2^ s^−1^). The two most attractive blue colors (Blue1 and Blue2) were then presented in a further dual-choice test. All LEDs were adjusted to the intensity of Blue1 (87.8 µmol m^−2^ s^−1^). The third experiment included the examination of the ultraviolet (UVA) spectral range by setting the four UV-A (UVA1-4) and the two violet LEDs (Violet1 and Violet2) to the same intensity (51.4 µmol m^−2^ s^−1^). Twenty replicates were performed for all multiple-choice tests and 10 replicates for dual-choice tests.

### 2.7. Test for Chromatic Interaction among Most Attractive Wavelengths: UV, Blue and Green

In the first experiment, six color traps were used. In order to investigate chromatic interactions, the basic colors Blue2, Green1, and UVA1, and all combinations of LEDs (UVA1+Blue2, UVA1+Green1, Blue2+Green1) were investigated. Green1 was used because it is a commercially available and affordable LED. Green2, used in the previous green-yellow spectral range experiment, is a very special and expansive multi-chip emitter which is present only once in the research group. All traps were each set to a total intensity of 44.5 µmol m^−2^ s^−1^. In the second experiment, four LED traps were used (Blue2, Green1, Blue2+Green1-100, and Blue2+Green1-200). Green1, Blue2, and the combination (Blue2+Green1-100) were set to a total intensity of 40 µmol m^−2^ s^−1^, corresponding to 100% relative intensity. The combination (Blue2+Green1-200) was adjusted to a total intensity of 80 µmol m^−2^ s^−1^ (200% relative intensity). In the third experiment, five color traps were compared. In addition to basic Blue2 and Green1, three different relative mixtures of both were created. The mixtures refer to 25%, 50%, and 75% relative intensity of the blue LED relative to the total intensity and vice versa. All traps were set to a total intensity of 45.6 µmol m^−2^ s^−1^. The individual blue and green LEDs were adjusted according to the proportions. In order to assess the preference of *F. occidentalis* more efficiently, repetitions of the three experiments were adapted to the number of traps used, so that each trap occurred equally often at each position. The first experiment was replicated 20 times, the second one 12 times, and the third one 15 times.

### 2.8. Data Analysis and Statistics

The statistical evaluation of the influence of wavelengths on *F. occidentalis* was conducted with the program R [26]. The relative choice frequencies, i.e., the preference of thrips for different LED colors, were analyzed using generalized linear models (GLMs), assuming a quasi-Poisson distribution (count data with overdispersion) [27]. By using a deviation analyses (*F*-test, link function: “log”) we investigated whether the factor color had a significant influence on the number of insects that are oriented towards the traps. The temporal repetitions of experiments were included in the models as a block factors. Subsequent Tukey-type multiple comparisons at α = 0.05 using the R-package “lsmeans” [28] were conducted to clarify which wavelength differs from another (mean value differences) with regard to the relative choice frequencies. Raw data is available at Leibniz Universität Hannover research data repository [29].

## 3. Results

### 3.1. Attractiveness of Different Wavelengths from the Ultraviolet to the Visible Spectral Range (307–635 nm)

**Figure 3:**
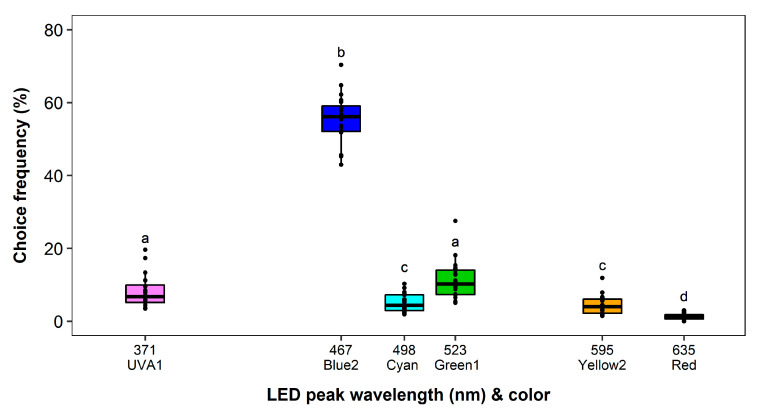
Wavelength preferences of *Frankliniella occidentalis* in multiple-choice experiment with light-emitting diodes (LEDs) in the complete spectral range of ultraviolet and visible light. See Table 1 and Figure 2 for LED color specifications. Significant differences are indicated by different letters (generalized linear model, Tukey test, *p* < 0.05, *n* = 20).

The recapture rate during the experiment was on average 85.76%. No block effect and a significant effect of the LED color on the choice frequency was observed (*F*_5,19_ = 240.34, *p* < 0.0001). The result showed a strong significant preference of *F. occidentalis* for Blue2 (55%) compared to the other colors used (*p* < 0.0001) (Figure 2). All other wavelengths attracted more than 5 times less thrips. UVA1 (8%) and Green1 (11%) were the second most attractive after Blue2, but did not differ in their attractiveness. Cyan (5%) and Yellow2 (4%) were of similar attractiveness and attracted significantly fewer thrips than UVA1 (*p* < 0.05) and Green1 (*p* < 0.0001). The attractiveness of Red was significantly the lowest compared to all other colors (1%, *p* < 0.01) (Figure 3).

**Figure 4:**
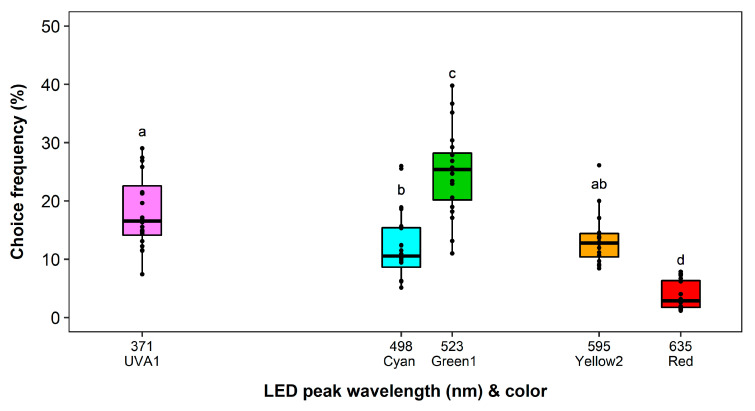
Wavelength preferences of *Frankliniella occidentalis* in multiple-choice experiment with light-emitting diodes (LEDs) in the complete spectral range of ultraviolet and visible light excluding blue. See Table 1 and Figure 2 for LED specifications. Significant differences are indicated by different letters (generalized linear model, Tukey test, *p* < 0.05, *n* = 20).

With exclusion of the most preferred color, i.e., Blue2, from the choice arena the overall recapture rate decreased slightly (72.27%). No block effect and a significant effect of the LED color on the choice frequency was observed (*F*_4,19_ = 38.97, *p* < 0.0001). The number of recaptured thrips on the remaining colors almost doubled, but the observed pattern was similar to the experiment before (Figure 4). However, Green1 (25%) was hereby significantly more attractive than UVA1 (18%, *p* = 0.0308).

### 3.2. Investigation of Thrips Visual Preference in the Green-Yellow (498–619 nm), Blue (413–498 nm), and Ultraviolet (371–413 nm) Spectral Ranges

**Figure 5:**
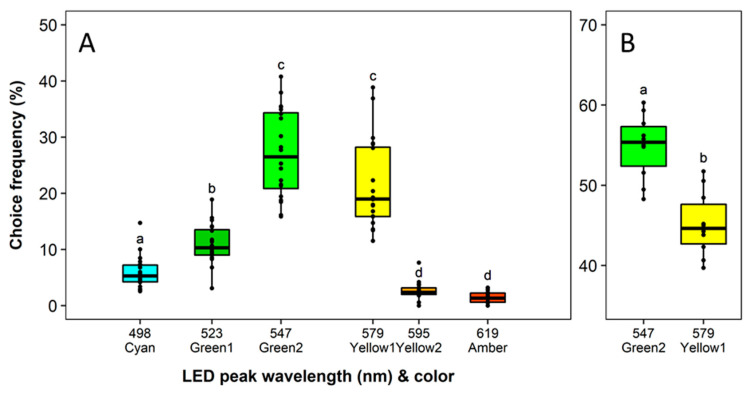
Wavelength preferences of *Frankliniella occidentalis* in multiple-choice experiment with light-emitting diodes (LEDs) in the spectral range from Cyan to Amber (**A**) and in dual-choice experiments with the two most attractive green and yellow LEDs (**B**). See Table 1 and Figure 2 for LED specifications. Significant differences are indicated by different letters (generalized linear model, Tukey test, *p* < 0.05, *n* = 20).

Overall recapture rates in the different experiments ranged from 45.64% to 72.97%. No block effect and a significant effect of the LED color on the choice frequency was observed in the green-yellow spectral range (*F*_5,19_ = 107.69, *p* < 0.0001). The attractiveness of the LED with the peak wavelength of 547 nm (Green2) was highest. The difference between the color traps Green2 and Yellow1 was not significant in the multiple-choice experiment. Both traps attracted approx. 2 times more thrips than all other traps (*p* < 0.0001) (Figure 5A). Green1 and Cyan followed in the preference order, with a significant difference between each other (*p* < 0.0024). Yellow2 and Amber were the least attractive, with comparable low numbers of recaptured thrips. In a further direct comparison of the most attractive colors Green2 (547 nm) and Yellow1 (579 nm), a significantly higher proportion of thrips was attracted to Green2 (*F*_1,9_ = 19.60, *p* < 0.0016) (Figure 5B).

**Figure 6:**
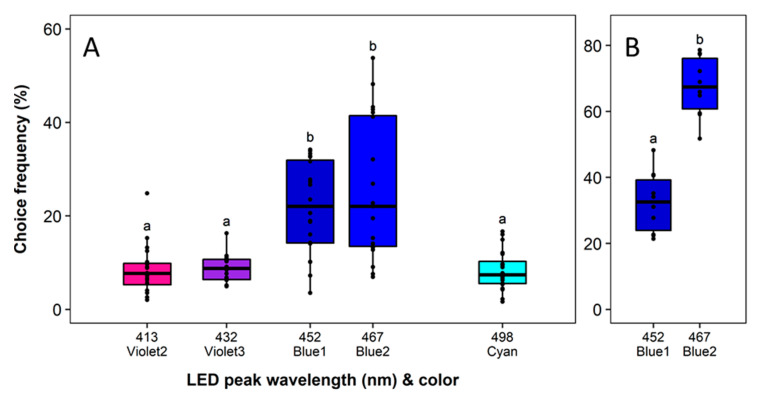
Wavelength preferences of *Frankliniella occidentalis* in multiple-choice experiment with light-emitting diodes (LEDs) in the spectral range from Violet to Cyan (**A**) and in dual-choice experiments with the two most attractive blue LEDs (**B**). See Table 1 and Figure 2 for LED specifications. Significant differences are indicated by different letters (generalized linear model, Tukey test, *p* < 0.05, *n* = 20).

No block effect and a significant effect of the LED color on the choice frequency was observed in the blue spectral range (*F*_4,19_ = 21.34, *p* < 0.0001). The attractiveness of the LED with the peak wavelength of 567 nm (Blue2) was highest but not significantly different from Blue1. Both traps attracted approx. 2 times more thrips than all other traps (*p* < 0.0001), which showed an equally low attractiveness (Figure 6A). In a further direct comparison of the most attractive colors Blue1 (452 nm) and Blue2 (467 nm), a significantly higher proportion of thrips was attracted to Blue2 (*F*_1,9_ = 35.07, *p* < 0.0001) (Figure 6B).

**Figure 7:**
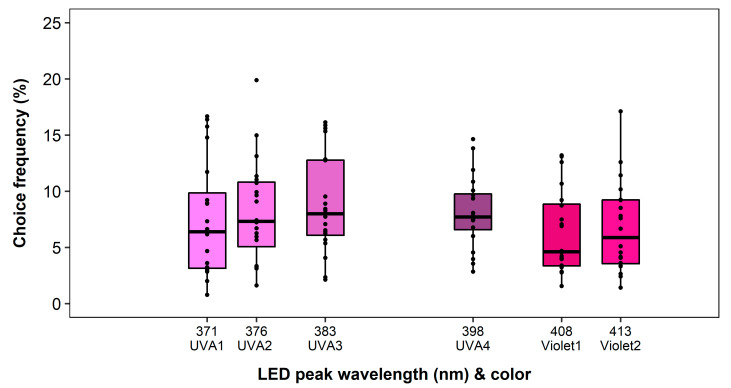
Wavelength preferences of *Frankliniella occidentalis* in multiple-choice experiment with light-emitting diodes (LEDs) in the ultraviolet and violet spectral range. See Table 1 and Figure 2 for LED specifications. No significant differences were observed (generalized linear model, *p* < 0.05, *n* = 20).

No block effect and no significant effect of the LED color on the choice frequency was observed in the ultraviolet to violet spectral range (*F*_5,19_ = 0.95, *p* = 0.4542). All tested traps showed similar attractiveness. The overall recapture rate was lowest among all experiments with 45.64%, while proportions of trapped thrips ranged between 6–9% on all traps (Figure 7).

### 3.3. Test for Chromatic Interaction among Most Attractive Wavelengths: UV, Blue, and Green

**Figure 8:**
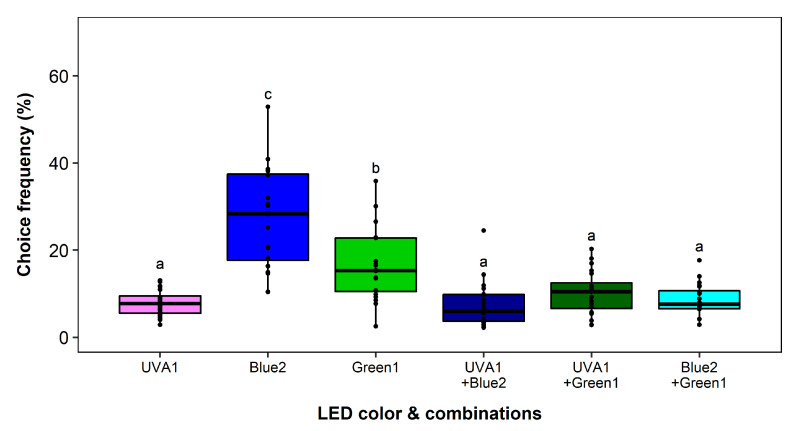
Wavelength preferences of *Frankliniella occidentalis* in multiple-choice experiment with ultraviolet, blue and green light-emitting diodes (LEDs) and respective combinations with adjusted equal intensities. See Table 1 and Figure 2 for LED specifications. Significant differences are indicated by different letters (generalized linear model, Tukey test, *p* < 0.05, *n* = 18).

Overall recapture rates in the different experiments ranged from 69.70% to 71.07%. At first, interactions between ultraviolet, blue, and green color were studied. No block effect and a significant effect of the LED color on the choice frequency was observed (*F*_5,19_ = 25.63, *p* < 0.0001). Blue2 and Green1 were the most attractive wavelengths, but differed significantly from each other (*p* = 0.0008) (Figure 8). In mutual combination and in combination with UVA1 (371 nm) the attractiveness of both colors was significantly lower than Green1 (*p* < 0.05) on the same level as UVA1 alone.

**Figure 9:**
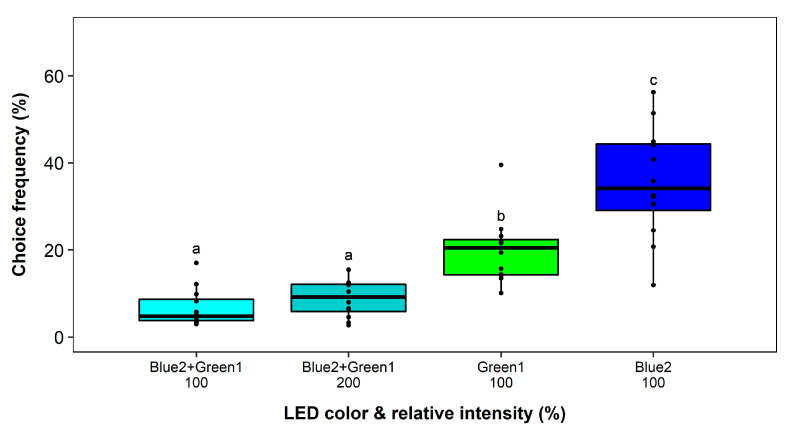
Wavelength preferences of *Frankliniella occidentalis* in multiple-choice experiment with blue and green light-emitting diodes (LEDs) and respective combinations with adjusted and additive intensities. See Table 1 and Figure 2 for LED specifications. Significant differences are indicated by different letters (generalized linear model, Tukey test, *p* < 0.05, *n* = 12).

**Figure 10:**
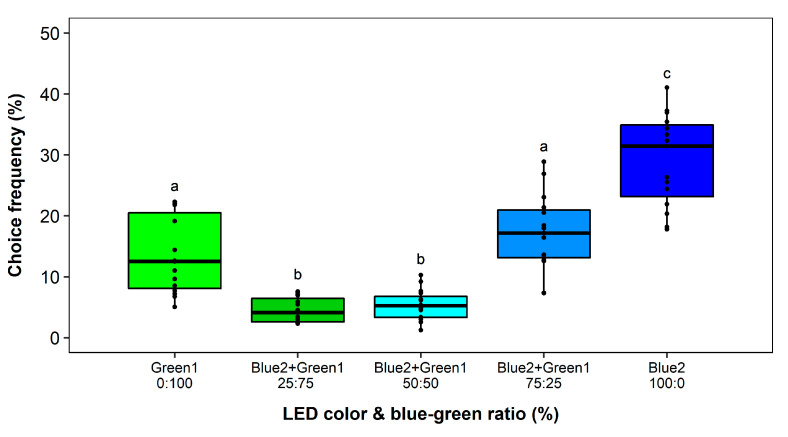
Wavelength preferences of *Frankliniella occidentalis* in multiple-choice experiment with blue and green light-emitting diodes (LEDs) and respective combinations with different intensity ratios. See Table 1 and Figure 2 for LED specifications. Significant differences are indicated by different letters (generalized linear model, Tukey test, *p* < 0.05, *n* = 15).

Finally, the interaction between blue and green color was investigated. No block effect and a significant effect of the LED color on the choice frequency was observed in the two experiments (*F*_3,11_ = 29.14, *p* < 0.0001; (*F*_4,14_ = 38.97, *p* < 0.0001)). Blue2 attracted significantly more thrips compared to Green1 in both experiments (Figure 9 and Figure 10). Moreover, the attractiveness dropped below 10% when blue and green LEDs were combined. Even with doubling of the light intensity, the attractiveness remained at the low level (Figure 9).

In contrast, increasing stepwise the proportion of the blue wavelength to the total intensity of the blue-green combination trap from 25% to 75% showed that already 25% of blue light resulted in a 3-fold reduction in attractiveness. With increasing the contribution of the blue light to 75%, the attractiveness was similar to green but still significantly lower compared to blue only (Figure 10).

## 4. Discussion

First of all, our results clearly show that a blue LED with a peak wavelength of 467 nm was most attractive for *F. occidentalis* compared to all other colors. This corroborates existing studies on the color preference of this species and the fact that blue sticky traps are widely used for monitoring and control in greenhouses [6,7,10,14]. In a previous study with a similar multiple-choice assay using the same light traps with comparable LEDs, two blue LEDs with peak wavelengths at 445 and 466 nm were identified as most attractive [10]. Due to the multiple-choice approach and the limited number of tested LEDs in that study, the two blue LEDs could not be properly separated, thus were regarded as equal in their attractiveness. Based on this screening, the shorter wavelength blue LED (445 nm) was selected for further experiments on the combination with blue sticky traps and olfactory attractants. Similarly in the present study, two blue LEDs (452, 467 nm) were not significantly different in their attractiveness in the multiple-choice experiment. However, a further dual-choice experiment revealed a significant preference for the 467 nm LED. For plant protection issues, blue LEDs around this peak wavelength can therefore be recommended to enhance the attractiveness of visual traps. Moreover, the color of (reflecting) card traps could potentially be modified according to the spectrum of this most attractive blue LED.

Our results also show that the green color trap with 523 nm peak wavelength was the second most attractive, and became most attractive when blue was excluded from the wide range multiple-choice setup. When the attractiveness of the green-yellow range was investigated in detail by presenting only colors ranging from Cyan to Amber, the green LED with 547 nm peak wavelength was most attractive, followed by a yellow LED (579 nm). This finding is in line with a similar study on the color choice behavior of the greenhouse whitefly *Trialeurodes vaporariorum*, where the same LED was most attractive [13]. Our results confirm the general fact that *F. occidentalis* is also attracted to the green-yellow range and can therefore be trapped on yellow sticky traps as well [2,14].

Although the blue preference of *F. occidentalis* is generally accepted [16], the literature on color preference is still ambiguous. For example, Ren et al. (2020) reported preferences of yellow followed by blue for laboratory conditions and blue followed by white for greenhouse conditions. An outdoor study in avocado orchards reported white as most attractive, followed by blue, yellow, and transparent traps, with a changing ratio of white and blue attractiveness over time [15]. This showed the difficulties of assessing color preferences in the field where many other internal and external factors could have affected the results. Blumthal et al. (2005) [30] highlighted that *F. occidentalis* preferred yellow flowers among others, i.e., white, orange, red, violet, and blue under laboratory conditions. The reported laboratory conditions comprise artificial lighting by fluorescent lamps, which provide a different spectrum compared to natural sunlight. It is likely that this setup has affected the results because the illumination spectrum is fundamental when studying color preferences with reflecting surfaces. With a higher ratio of yellow radiation from the fluorescent lamps, yellow in particular appears much brighter compared to blue. Therefore, changing preference could be explained since intensity dependence within the attractive blue range was reported for *F. occidentalis* [10].

Moreover, studies with broadband reflection patterns of colored stimuli commonly do not clearly separate intensity and wavelength-dependent responses. The use of narrow bandwidth LEDs at equal intensities overcomes these drawbacks and our results can be interpreted purely based on the wavelength patterns. Therefore, our study provides behavioral evidence that *F. occidentalis* is generally attracted to two separate spectral ranges, suggesting two separate wavelength-specific behaviors. This can be explained only by the presence of photoreceptors with maximal sensitivity in the blue and green range. Nevertheless, this hypothesis is in contrast to the only available physiological study, which highlights spectral sensitivities only in the UV-A (365 nm) and green (540 nm) range [17]. Matteson et al. (1992) explained the phenomenon of the blue preference in *F. occidentalis* by the fact that the blue wavelength range could be perceived by simultaneous excitation of the green and UV photoreceptors. However, this can be clearly ruled out according to our current results.

Wavelength-specific behaviors rely on photoreceptors with different sensitivities. They can drive behaviors independent from each other or the outputs inhibit each other, either on a neuronal level or in the motor output. A behavior which is controlled only by one receptor is regarded as color blind, while an inhibitory interaction between two receptors with overlapping sensitivities enables the extraction of chromatic signals which in general allows color vision [19]. Color vision can be defined as the ability to detect spectral variations in the light independent of their intensity [20]. Therefore, our results regarding combinations of blue and green LED light give compelling indications for a chromatic interaction between two photoreceptors in the green and blue range. Combining blue and green light decreased the attractiveness significantly compared to blue or green light alone. This was also the case when the intensity of both lights in the combination was doubled. The attractiveness increased again when the relative amounts of the respective green and blue light was shifted stepwise towards the one or the other wavelength. Such an inhibitory interaction, referred to as “blue-green opponency” or “opponent mechanism”, is well accepted for other herbivorous insects. Here the signal from the green receptor is excitatory, while the signal from the blue receptor is inhibitory [22,23]. In other words, a kind of non-behavior is elicited by the blue receptor output, which results in a repellence by the blue spectral range, which cannot be measured as a behavior in choice experiments. While in an LED choice study with the greenhouse whitefly, a blue LED with 467 nm peak wavelength was most inhibitory for the settling behavior [13], the same LED was most attractive for *F. occidentalis* in the present study. This supports the conclusion that a second behavior is obviously elicited by the blue photoreceptor, which can be measured as attraction in choice experiments. Nevertheless, a mutual interaction seems to be present as already discussed.

Since Matteson et al. (1992) only found a peak efficiency around 540 nm in an electrophysiological study, we can assume that the peaks of the underlying blue and green photoreceptors might be quite close together. Most likely the sum of the signals from the two receptors was measured in the electroretinogram recordings [16]. Similarly, the presence of two receptors went unnoticed in electrophysiological studies of only white- or dark-adapted eyes of whiteflies, aphids, and the pollen beetle [23,31,32]. This contradicts behavioral choice data as well and could only be estimated by empirical modeling or LED light combination experiments [13,22,23]. Inhibitory interactions between photoreceptors generally shift the behavioral action spectra as compared to the sensitivity of the underlying photoreceptor [17,19]. In combination with the spectral efficiency data [16], it can be assumed from our results that both receptor peaks are very close together and might actually both be located close to the blue-green range around 500 nm. However, final conclusions on the exact or approximate photoreceptor positions cannot be given and further physiological or modeling-based investigations are required in the future.

But what are the ecological implications for *F. occidentalis* showing wavelength-specific behaviors towards the blue and green ranges? Regarding the green range the explanation is straight forward, since thrips feed and oviposit on green leaf tissue. Plant leaves have a reflection peak around 550 nm similar to the most attractive green LED (547 nm). Consequently, our results can be explained by the fact that the visual system is adapted to host plant detection for feeding and oviposition, as already known for other leaf-feeding insects [13,17,18,33].

Because the western flower thrips feeds also on pollen and nectar, it can be speculated that the attraction to blue is caused by an adaptation to floral resources. Interestingly, the onion fly *Delia antiqua* and the cabbage butterfly *Pieris rapae* show similar behavior to *F. occidentalis* in terms of color preferences, since blue-colored traps were preferred to others. For both insects, the authors assume that the blue spectral range could possibly trigger a wavelength-specific landing or feeding behavior [34,35,36]. Since *D. antiqua* and *P. rapae* feed mainly on pollen and nectar and most of the flowers of their hosts reflect a high proportion of violet-blue light, the preference and behavior could thus be explained [37]. In contrast, *F. occidentalis* is a polyphagous insect without preference for specific host plants with blue-violet flowers [38]. Nevertheless, the relevance of the flowers in relation to the population dynamics of the thrips is prevalent. For example, Yudin et al. (1988) [39] already showed that more thrips are found on flowers than on the vegetative plant tissue. 

Therefore, it is likely that stimulation of behavior by green and blue is similar in its motoric processing, but the purpose of the behavior is different. Similarly, in *P. brassicae* the individual spectral regions each trigger a specific behavior, i.e., blue triggers feeding and green oviposition, but become less attractive when both wavelengths are perceived simultaneously [40]. However, the complete ecological reasons for the blue preference in *F. occidentalis* cannot be explained from our results and further investigations are needed in the future. Moreover, it is possible that this preference will slightly change during the life cycle. We used only lab-reared thrips of mixed sex which freshly emerged from the pupation site. The situation might be different for wild thrips or for individuals at a different physiological status, e.g., older well-fed female thrips with high egg-load which search for oviposition sites on green leaves. Slight differences between males and females are likely as well, but were not regarded in this study.

For UV LEDs only a moderate attractiveness slightly below the attractiveness of green was observed throughout when presented with blue and/or green in the multiple-choice setup, which supports our previous study [10]. In addition, the combination of UV with blue and green did not show any potential to increase the attractiveness. This suggests that their use in visual traps for plant protection seems not very advantageous.

Nevertheless, the results confirm the general sensitivity for UV and leave no doubt about the presence of a UV-sensitive photoreceptor [16]. The response to UV is based on a behavioral pattern which can be broadly related to dispersal behavior in *F. occidentalis* [24], and some kind of migratory behavior could have been elicited by the UV taps [40]. However, no differentiation in terms of attractiveness could be observed when different LEDs from UV to the lower wavelength violet range were presented. This could be explained by a broad sensitivity or an oversaturation of a receptor in this range and indicates the absence of any chromatic interaction. The fluorescent lamps in the climate chamber did not provide much UV and the intensity emitted from the traps was relatively high. The behavior was probably stimulated to an equal degree by all traps, which is in contrast to a similar study with whiteflies under greenhouse conditions [13]. Nevertheless, it could be observed that the behavior towards UV was substantially different compared to the directional response to blue and green. The overall recapture rate was lowest in this experiment and the remaining thrips were distributed all over the arena.

## 5. Conclusions

*Frankliniella occidentalis* most likely exhibits two, probably different wavelength-specific behaviors towards the blue and green spectral range. Our results provide compelling indications that these behaviors are controlled by two photoreceptors maximally sensitive for blue and green with an inhibitory chromatic interaction between both. The presence of a UV-sensitive photoreceptor could be confirmed. Consequently, a trichromatic photoreceptor setup is suggested for *F. occidentalis*. Nevertheless, the obtained results are exclusively limited to the thrips used in the experiments, i.e., freshly emerged lab-reared adult thrips of mixed sex. The color preferences need to be validated and specified for wild thrips at different physiological states and known sex ratios in future studies.

Concerning advanced plant protection strategies, it can be concluded that blue and green LEDs can be used to enhance the attractiveness of visual traps for *F. occidentalis*. Blue LEDs are thereby the most attractive, but also a very species-specific target. Considering the common green-yellow attractiveness to other pest insects and natural enemies, green LEDs would be the alternative for more general traps. Their potential advantage compared to colored sticky traps should be evaluated in the future.

## Figures and Tables

**Table 1:** Specifications of high-power light-emitting diodes (LEDs) and constructed panels used.

LED Colour Names	Peak Wavelength (nm)	Manufacturer	Type (Design *)	LEDs/Panel (Cooling **)
UVA1	371	Roithner	H2A1-H365-E (sc)	1 (nc)
UVA2	376	Roithner	H2A1-H375-E (sc)	1 (nc)
UVA3	383	Roithner	H2A1-H385 (sc)	1 (nc)
UVA4	398	Roithner	H2A1-H395 (sc)	1 (nc)
Violet1	408	Roithner	H2A1-H405 (sc)	1 (nc)
Violet2	413	Roithner	H2A1-H410 (sc)	1 (nc)
Violet3	432	Roithner	H2A1-435 (sc)	1 (nc)
Blue1	452	Cree	XP-E (sc)	1 (nc)
Blue2	467	Osram	Oslon SSL LB CP7P (sc)	1 (nc)
Cyan	498	Roithner	H2A1-490 (sc)	1 (nc)
Green1	523	Roithner	H2A3-520 (sc)	1 (nc)
Green2	547	Roithner	LED550-66-60 (mc)	1 (pc)
Yellow1	579	Roithner	LED570-66-60 (mc)	4 (ac)
Yellow2	595	Osram	Oslon SSL LY CP7P (sc)	2 (nc)
Amber	619	Osram	Oslon SSL LA CP7P (sc)	1 (nc)
Red	635	Nichia	NCSR119 T (sc)	1 (nc)

* sc = single chip emitter, mc = multi chip emitter, ** nc = no additional cooling, pc = passive cooling with heat sink, ac = active cooling with fan.
